# Identification and characterization of a Golgi retention signal in feline coronavirus accessory protein 7b

**DOI:** 10.1099/jgv.0.000879

**Published:** 2017-07-31

**Authors:** Dominik Florek, Rosina Ehmann, Claudia Kristen-Burmann, Tanja Lemmermeyer, Günter Lochnit, John Ziebuhr, Heinz-Jürgen Thiel, Gergely Tekes

**Affiliations:** ^1^​ Institute of Virology, Justus Liebig University Giessen, Germany; ^2^​ Institute of Biochemistry, Justus Liebig University Giessen, Germany; ^3^​ Institute of Medical Virology, Justus Liebig University Giessen, Germany; ^†^​ Present address: JOTEC GmbH, Lotzenäcker 23, 72379 Hechingen, Germany.

**Keywords:** feline coronavirus, feline infectious peritonitis, coronavirus accessory proteins, reverse genetics, Golgi retention, cellular trafficking

## Abstract

Feline coronaviruses encode five accessory proteins with largely elusive functions. Here, one of these proteins, called 7b (206 residues), was investigated using a reverse genetic approach established for feline infectious peritonitis virus (FIPV) strain 79–1146. Recombinant FIPVs (rFPIVs) expressing mutant and/or FLAG-tagged forms of 7b were generated and used to investigate the expression, processing, glycosylation, localization and trafficking of the 7b protein in rFIPV-infected cells, focusing on a previously predicted ER retention signal, KTEL, at the C-terminus of 7b. The study revealed that 7b is N-terminally processed by a cellular signalase. The cleavage site, 17-Ala|Thr-18, was unambiguously identified by N-terminal sequence analysis of a 7b processing product purified from rFIPV-infected cells. Based on this information, rFIPVs expressing FLAG-tagged 7b proteins were generated and the effects of substitutions in the C-terminal _202_KTEL_206_ sequence were investigated. The data show that (i) 7b localizes to and is retained in the *medial*- and/or *trans*-Golgi compartment, (ii) the C-terminal KTEL sequence acts as a Golgi [rather than an endoplasmic reticulum (ER)] retention signal, (iii) minor changes in the KTEL motif (such as KTE, KTEV, or the addition of a C-terminal tag) abolish Golgi retention, resulting in relocalization and secretion of 7b, and (iv) a KTEL-to-KDEL replacement causes retention of 7b in the ER of rFIPV-infected feline cells. Taken together, this study provides interesting new insights into an efficient Golgi retention signal that controls the cellular localization and trafficking of the FIPV 7b protein in virus-infected feline cells.

## Introduction

Feline coronaviruses (FCoVs) are enveloped viruses with a positive-strand RNA genome of approximately 30 kb. FCoVs are members of the family *Coronaviridae* that, together with the families *Arteriviridae*, *Mesoniviridae* and *Roniviridae*, form the order *Nidovirales* [1]. The subfamily *Coronavirinae* within the family *Coronaviridae* is divided into four genera: *Alpha-*, *Beta-*, *Gamma-* and *Deltacoronavirus*. Feline coronaviruses, canine coronaviruses (CCoV) and porcine transmissible gastroenteritis virus (TGEV) are closely related and have therefore been assigned to the same species, called *Alphacoronavirus 1*, within the genus *Alphacoronavirus*. More distantly related species in the genus *Alphacoronavirus* include porcine epidemic diarrhoea virus (PEDV), human coronavirus 229E (HCoV-229E) and human coronavirus NL63 (HCoV-NL63) [2].

FCoVs infect both domestic and wild *Felidae*. They are widespread in the cat population worldwide, with seropositivity rates approaching 90 % in animal shelters and multi-cat households [3, 4]. FCoVs are divided into two serotypes [5–7]. While serotype I FCoVs are responsible for more than 90 % of the infections, serotype II FCoVs are less frequent and mainly occur in Asia [3, 8]. Serotype II FCoVs evolved by a double homologous recombination between serotype I FCoVs and CCoVs [9, 10].

Both serotypes exist in two distinct biotypes that are also referred to as ‘pathotypes’. While feline enteric coronavirus (FECV) causes asymptomatic persistent infections of the gut, feline infectious peritonitis virus (FIPV) causes a lethal disease called feline infectious peritonitis (FIP) [4]. According to the current view in the field, FIPV evolves from FECV in persistently infected cats by acquiring mutations in the viral genome [4, 11–14]. To date, the mutations responsible for the biotype switch have not been unambiguously identified, but there is now strong evidence from comparative sequence analyses of paired virus samples to suggest that genetic changes in the S gene and, to a lesser extent, in the accessory genes are critically involved in the conversion between the two biotypes, FECV and FIPV [4, 11, 12, 15–19].

Coronaviruses contain varying numbers of accessory genes in their 3′-proximal genome regions. Accessory gene products are generally thought to represent virulence factors, but, with few exceptions, such as SARS-CoV, the precise functions of coronavirus-encoded accessory proteins have not been characterized in detail [20–22]. Interestingly, the majority of accessory protein genes are only conserved in closely related coronaviruses, suggesting a relatively recent acquisition and distinct functions of these proteins in facilitating viral replication in specific ecological niches [21–23]. Similar to other alphacoronaviruses, the accessory genes of FCoV are located in two genome regions, (i) between the S and E genes and (ii) downstream of the N gene. FCoVs and CCoVs share three ORFs (3a, 3b and 3c) in the region between S and E, whereas TGEV possesses only two ORFs (3a and 3b) in this genome region. Furthermore, an additional ORF (ORF 3), which encodes a glycoprotein, gp3, was identified in this genome region in CCoV type 1, while only remnants of this gene were identified in other viruses of the species *Alphacoronavirus 1* [24–26]. Several other alphacoronaviruses, such as PEDV, HCoV-229E and HCoV-NL63, only harbour one accessory protein gene in this genome region (called ORF 3 in PEDV and HCoV-NL63 and ORF 4 in HCoV-229E, respectively). Comparative sequence analyses revealed that FCoV ORF 3 c has homologues in (all) other alphacoronaviruses that are currently known (called ORF 3c in CCoV, ORF 3b in TGEV, ORF 3 in PEDV and HCoV-NL63, and ORF4 in HCoV-229E) [2]. Furthermore, homologues of FCoV ORF 3a are conserved in both CCoV and TGEV, while the FCoV ORF 3b is conserved in CCoV but not in TGEV. Between the N gene and the 3′ UTR, the FCoV and CCoV genomes have two ORFs (7a and 7b), while that of TGEV contains only one ORF, called ORF 7. The latter encodes a protein that is homologous to the gene products encoded by the ORF 7as of FCoV and CCoV, respectively. The ORF 7bs of FCoV and CCoV encode homologous proteins that have no known counterparts in other coronaviruses.

Critical roles for ORF 3/7-encoded proteins in viral pathogenesis have been demonstrated by using ORF 3abc and 7ab deletion mutants derived from FIPV (strain 79–1146) [27], but the precise functions of the individual accessory proteins in FCoV pathogenesis remain to be elucidated. It has been suggested that the ORF 3c-encoded protein is important for replication in the intestine but is, most likely, dispensable for systemic spread [16]. For the FCoV 7a gene product, a role in counteracting IFNα-induced antiviral responses has been proposed [28], but the production of this protein in infected cells remains to be formally demonstrated. Initial information on the FCoV 7b protein was obtained in an *in vitro* study published in the early 1990s [29]. The protein (called the 6b protein in this earlier study) was detected in FCoV-infected cells as a 26.5 kDa protein using sera obtained from naturally infected animals [29]. Further studies using a vaccinia virus/T7-based system to produce 7b in HeLa cells revealed that 7b is a soluble glycoprotein that primarily resides in the ER lumen but may also be secreted, albeit relatively slowly, into the cell culture supernatant. The very C-terminal KTEL motif present in 7b was identified as a KDEL-like ER retention signal. A Leu-to-Val substitution in the KTEL motif (KTEV) abolished 7b retention and facilitated secretion of the protein [29]. A Thr-to-Asp exchange resulting in a KDEL motif led to complete ER retention of 7b. Moreover, an N-terminal hydrophobic sequence was suggested to act as a signal sequence [29].

In this study, we employed a set of recombinant FCoVs produced by reverse genetics to obtain new insights into the expression, localization and posttranslational modification of the 7b protein, and the role of the C-terminal KTEL motif in cellular trafficking in FCoV-infected feline cells.

## Results

### Subcellular localization of accessory protein 7b in FCoV-infected cells

**Fig. 1.**
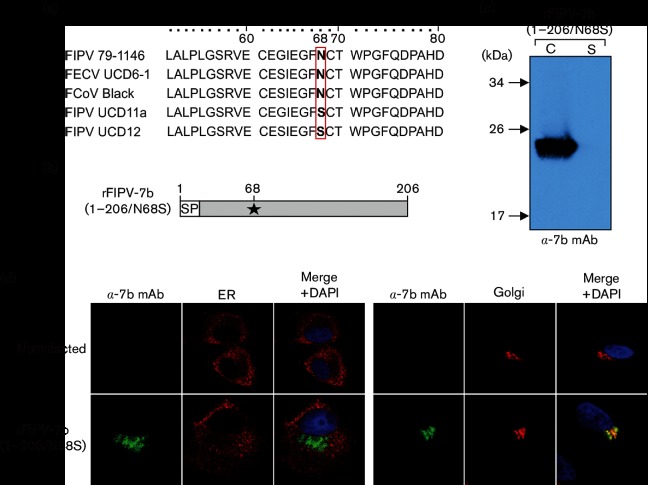
Detection of accessory protein 7b in FIPV-infected cells by anti-7b monoclonal antibody 14D8 (α−7b mAb). (a) Sequence alignment of amino acids (aa) 51–80 in 7b. FIPV 79–1146 (AY994055), FECV UCD6-1 (ACR46154), FCoV Black (EU186072), FIPV UCD11a (ACR10287.1) and FIPV UCD12 (ACR10305.1). AA position 68 is highlighted. (b) Schematic representation of the 7b protein encoded by rFIPV-7b(1–206/N68S). The Asn-to-Ser substitution at position 68 (N68S) is indicated by an asterisk. SP, signal peptide; numbers indicate amino acid positions in 7b. The arrow represents the predicted signalase cleavage site between residues 17 and 18. (c) CRFK cells were infected with rFIPV-7b(1–206/N68S)at an m.o.i. of 0.1. Cells and cell culture supernatant were harvested at 16 h p.i. Cell lysate (C) and cell culture supernatant (S) were separated by SDS-PAGE (10 %) under reducing conditions and analysed by Western blotting using anti-7b mAb. (d) CRFK cells were mock-infected or infected with rFIPV-7b(1–206/N68S)at an m.o.i. of 1. Cells were fixed at 16 h p.i. and analysed by confocal laser-scanning microscopy. Immunofluorescence staining of 7b was performed with anti-7b mAb (green signal). Immunofluorescence staining of the endoplasmic reticulum (ER) (left panel, red signal) and the Golgi complex (right panel, red signal) is shown. Cell nuclei (blue signal) were stained with DAPI.

In a previous study, we reported on the generation of mAbs against the FCoV 7b protein [30]. The mAbs obtained in this study detected the 7b protein in its non-glycosylated precursor form, but unfortunately failed to detect the mature, glycosylated form of the 7b protein. Accordingly, interesting aspects of 7b, such as the subcellular localization in wild-type FCoV-infected cells, and secretion of the protein from these cells, could not be studied using these mAbs. To resolve this technical problem, we generated a recombinant FCoV that encodes a 7b protein that lacks the single predicted N-glycosylation site (Asn-68). Analyses of 7b sequences available in the database revealed that some field isolates (FIPV UCD 11a, GenBank ACR10287.1; FIPV UCD12, GenBank ACR10305.1) carry Ser instead of Asn at this position (Fig. 1a), suggesting that N-glycosylation of Asn-68 is not essential and an Asn-to-Ser substitution at this position will be tolerated very well. Replacement of the Asn-68 codon with a Ser codon in ORF7b (N68S) resulted in rFIPV-7b(1–206/N68S) (Fig. 1b). The virus was used to analyse the subcellular localization of 7b in rFIPV-7b(1–206/N68S)-infected CRFK cells. At 16 h p.i. [i.e. in the absence of a detectable cytopathic effect (CPE)], cell culture supernatant and cell lysate were collected and subjected to Western blot analysis using anti-7b mAb 14D8 (Fig. 1c). In cell lysates obtained from virus-infected cells, a protein corresponding to the predicted molecular mass of FCoV 7b (~24 kDa) was readily detected, suggesting that the non- glycosylated 7b_N68S protein was efficiently expressed in rFIPV-7b(1–206/N68S)-infected cells; there was no evidence for secretion of this protein into the supernatant (Fig. 1c). To determine the subcellular localization of 7b, CRFK cells were infected with rFIPV-7b(1–206/N68S), fixed at 16 h p.i., and analysed by confocal microscopy. Interestingly, the 7b protein was found to co-localize with the Golgi apparatus, while there was no evidence for an ER localization (Fig. 1d). Together, these data suggest that the non-glycosylated form of 7b (7b_N68S) is retained in the Golgi apparatus and not secreted from infected cells. The data contradict conclusions from previous studies that were based on vaccinia virus/T7-based expression systems and suggested that the 7b protein would (i) primarily localize to the ER and (ii) be secreted in the supernatant [29].

### Recombinant FCoVs expressing FLAG-tagged 7b proteins

**Fig. 2.**
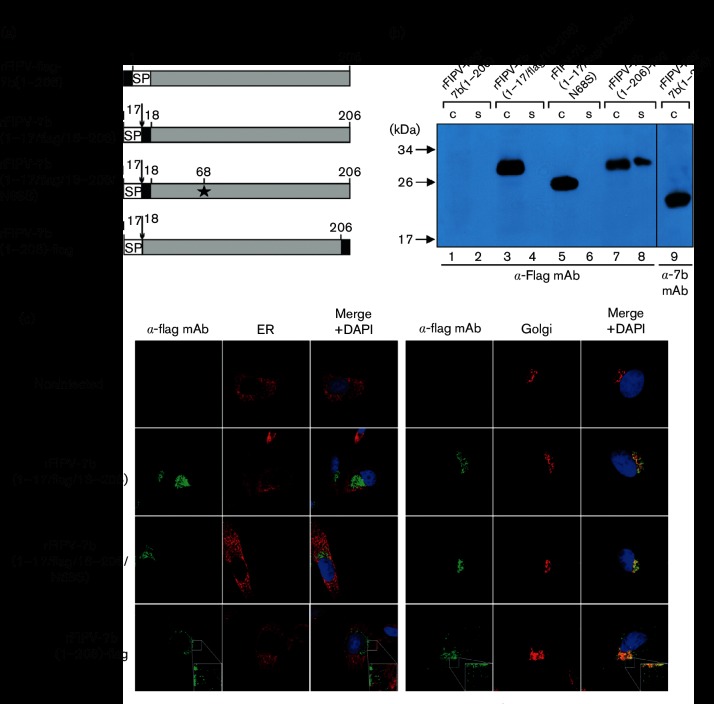
Detection of 7b accessory protein in FIPV-infected cells. (a) Schematic representation of 7b protein variants encoded by rFIPV-FLAG-7b(1-206), rFIPV-7b(1–17/FLAG/18–206), rFIPV-7b(1–17/FLAG/18–206/N68S) and rFIPV-7b(1-206)-FLAG, respectively. Black boxes represent the positions of the FLAG tag in the respective 7b protein variants. The Asn-to-Ser substitution at position 68 (N68S) is indicated by an asterisk. SP, signal peptide; numbers indicate amino acid positions in 7b. The arrow represents the signalase cleavage site. (b) CRFK cells were infected with rFIPV-FLAG-7b(1-206), rFIPV-7b(1–17/FLAG/18–206), rFIPV-7b(1–17/FLAG/18–206/N68S) and rFIPV-7b(1-206)-FLAG, respectively, with an m.o.i. of 0.1. Cells and cell culture supernatants were harvested at 16 h p.i. Cell lysates (C) and cell culture supernatants (S) were separated by SDS-PAGE (10 %) under reducing conditions and analysed by Western blotting using anti-FLAG mAb. Further, a cell lysate obtained from rFIPV-FLAG-7b(1-206)-infected cells was analysed by Western blotting using anti-7b mAb 14D8 (α−7b mAb). Lanes are numbered (1–9) at the bottom of the figure. (c) CRFK cells were mock-infected or infected with rFIPV-7b(1–17/FLAG/18–206), rFIPV-7b(1–17/FLAG/18–206/N68S) and rFIPV-7b(1-206)-FLAG, respectively, with an m.o.i. of 1. Cells were fixed at 16 h p.i. and analysed by confocal laser-scanning microscopy. Immunofluorescence staining of 7b was performed using anti-FLAG mAb (α-FLAG mAb, green signal). Immunofluorescence staining of the endoplasmic reticulum (ER) (left panel, red signal) and the Golgi complex (right panel, red signal) is shown. Cell nuclei were stained with DAPI (blue signal). The inserts in the lower right corner represent an 8× magnification of the selected area.

To exclude the possibility that the features described above for 7b_N68S were due to the absence of the glycosylation site, or possibly other effects caused by the Asn-to-Ser substitution at position 68, we generated a set of viruses that expressed 7b proteins with an intact N-glycosylation site (Asn-68). In these cases, however, a FLAG tag was fused to the 7b sequence to facilitate protein detection using FLAG tag-specific mAbs. First, a virus called rFIPV-FLAG-7b(1-206) was produced. The recombinant virus was designed to express a 7b protein with an N-terminal FLAG tag. As shown in Fig. 2(a), the N-terminally FLAG-tagged 7b protein could not be detected in lysates and supernatants obtained from rFIPV-FLAG-7b(1-206)-infected cells, while a clear signal for a~24 kDa protein expressed in these cells was obtained if the anti-7b mAb 14D8 was used (Fig. 2b, lanes 1, 2 and 9). The size of the 24 kDa protein is consistent with the molecular mass calculated for the non-glycosylated form of 7b. These data and previous predictions for a potential signal sequence located in the N-terminal region of 7b [29] led us to conclude that the N-terminally FLAG-tagged 7b protein was efficiently expressed in virus-infected cells but, due to signalase-mediated processing, the N-terminal sequence including the FLAG tag had been removed, thus preventing detection of the mature protein using the FLAG-specific mAb.

*In silico* predictions using the SignalP 4.1 server [31] revealed a potential signalase cleavage site between amino acid positions 17 and 18 in the 7b protein and another (albeit less probable) cleavage site between 19 and 20 (_15_IKA^↓^TA^(↓)^VQN_22_). To determine the signalase cleavage site unambiguously, we infected CRFK cells with rFIPV-7b(1–206/N68S), which encodes the non-glycosylated form of 7b, and immunoprecipitated the N-terminally processed 7b protein from cell lysates prepared at 16 h p.i. using the anti-7b mAb. The precipitated 7b protein was separated by SDS-PAGE, blotted onto a PVDF membrane, and stained. The band corresponding to the non-glycosylated form of 7b (24 kDa) was excised and used for N-terminal sequence analysis by Edman degradation. The sequencing data obtained for seven amino-terminal residues revealed that cleavage occurs between aa 17 and 18 of the 7b protein (data not shown).

Based on this information, our further studies of the expression, intracellular localization and secretion of 7b were based on recombinant viruses that expressed 7b proteins with a FLAG tag inserted immediately downstream of the signalase cleavage site. This included rFIPV-7b(1–17/FLAG/18–206) and a virus called rFIPV-7b(1–17/FLAG/18–206/N68S). The latter is identical to rFIPV-7b(1–17/FLAG/18–206), but contains an Asn-to-Ser exchange at aa position 68 to prevent N-linked glycosylation of 7b (Fig. 2a). Using these viruses, cell culture supernatants and cell lysates were obtained from infected CRFK cells and analysed by Western blotting. In both cell lysates, 7b could be detected with anti-FLAG mAb. As expected, the 7b protein expressed in rFIPV-7b(1–17/FLAG/18–206/N68S)-infected cells migrated faster in the gel than the 7b protein expressed in rFIPV-7b(1–17/FLAG/18–206)-infected cells, due to the absence of N-glycosylation in the FLAG-tagged 18–206_N68S protein (Fig. 2b). Secretion of 7b could not be observed in any of the cell culture supernatants, indicating that the intracellular retention of 7b in infected cells is independent of the glycosylation state. To determine the subcellular localization of glycosylated and non-glycosylated forms of 7b, confocal microscopy analyses were performed (Fig. 2c). CRFK cells were infected with rFIPV-7b(1–17/FLAG/18–206) and rFIPV-7b(1–17/FLAG/18–206/N68S), respectively, and fixed 16 h p.i. The analyses with anti-FLAG mAb showed that both the glycosylated and non-glycosylated forms of 7b co-localized exclusively with the Golgi apparatus, suggesting that the glycosylation state of the 7b protein did not affect the intracellular localization of the protein. Furthermore, the subcellular localization of FLAG-tagged 7b was identical to that of the non-FLAG-tagged form expressed in rFIPV-7b(1–206/N68S)-infected cells (Fig. 1a), suggesting that the insertion of the FLAG tag downstream of aa 17 is a suitable approach for the further characterization of the 7b protein.

### Retention of 7b beyond the *cis*-Golgi compartment

**Fig. 3.**
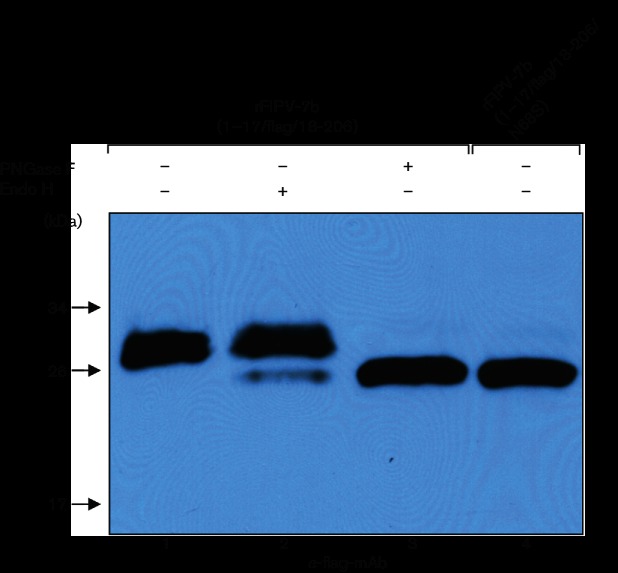
Glycosidase treatment of accessory protein 7b. CRFK cells were infected with rFIPV-7b(1–17/FLAG/18–206 and rFIPV-7b(1–17/FLAG/18–206/N68S), respectively, with an m.o.i. of 0.1. Cell lysates obtained at 16 h p.i. were subjected to glycosidase (PNGase F or Endo H) treatment (+) or left untreated (−) as indicated. Subsequently, cell lysates were separated by SDS-PAGE (10 %) under reducing conditions and analysed by Western blotting using anti-FLAG mAb (α-FLAG mAb). Lanes are numbered (1–4) at the bottom of the figure.

To determine the compartment of the Golgi apparatus where 7b is retained, CRFK cells were infected with rFIPV-7b(1–17/FLAG/18–206) and the cell lysate prepared from these cells was subjected to endoglycosidase H (Endo H) and PNGase F glycosidase treatment, respectively, prior to Western blot analysis (Fig. 3). PNGase F treatment, which is known to remove all oligosaccharides from Asn-linked glycoproteins, resulted in a~26 kDa protein that co-migrated with the non-glycosylated (N68S) N-terminally FLAG-tagged form of the 7b protein (Fig. 3, lanes 3 and 4). If the lysate was treated with Endo H, Endo H-sensitive and Endo H-resistant forms of the FLAG-tagged 7b protein were detected with apparent molecular masses of ~26 and~28 kDa, respectively. The major fraction of 7b was Endo H-resistant, indicating that this form of 7b contains complex glycan structures rather than asparagine-linked high-mannose oligosaccharide side-chains. Since complex glycan structures are known to be formed beyond the *cis*-Golgi, the data lead us to conclude that the major part of 7b is localized in the *medial*- and/or *trans*-Golgi compartments. The (minor) Endo H-sensitive 7b fraction probably represents newly synthesized and incompletely glycosylated forms of 7b that have not yet arrived at the *medial*-Golgi compartment.

### Critical role of the C-terminal tetrapetide sequence in the subcellular localization of the 7b protein

In a recent publication, the production of a 7b–GFP fusion protein in FCWF-4 cells was described [28]. Consistent with the data described above, the 7b–GFP fusion protein was found to localize to the Golgi apparatus. However, a major part of the 7b–GFP protein localized to dense spots throughout the cytoplasm, which were not detected in FIPV-infected cells expressing a 7b protein with an authentic C-terminus (Fig. 2). To answer the question of whether additional C-terminal sequences affect the subcellular localization of the 7b protein in FCoV-infected cells, we generated a virus, rFIPV-7b(1-206)-FLAG, in which a FLAG tag was fused to the C-terminus of 7b. CRFK cells were infected with this virus, and cells and cell culture supernatant were collected at 16 h p.i. and subjected to Western blot analysis. As shown in Fig. 2(b) (lanes 7 and 8), the C-terminally FLAG-tagged 7b protein could be detected in both infected cells and the supernatant (Fig. 2b), while 7b proteins with a FLAG tag downstream of the signalase cleavage site were not detected in cell culture supernatants (Fig. 2, lanes 4 and 6). Confocal microscopy analyses revealed that C-terminally FLAG-tagged 7b co-localized with the Golgi apparatus but also produced dense spots in the cytoplasm without any association with the Golgi (Fig. 2c), which is very similar to what has been described for the 7b–GFP fusion protein discussed above [28]. These data suggest that artificial C-terminal sequences, such as a FLAG tag or GFP, affect the subcellular localization of 7b and lead to secretion of the protein.

**Fig. 4.**
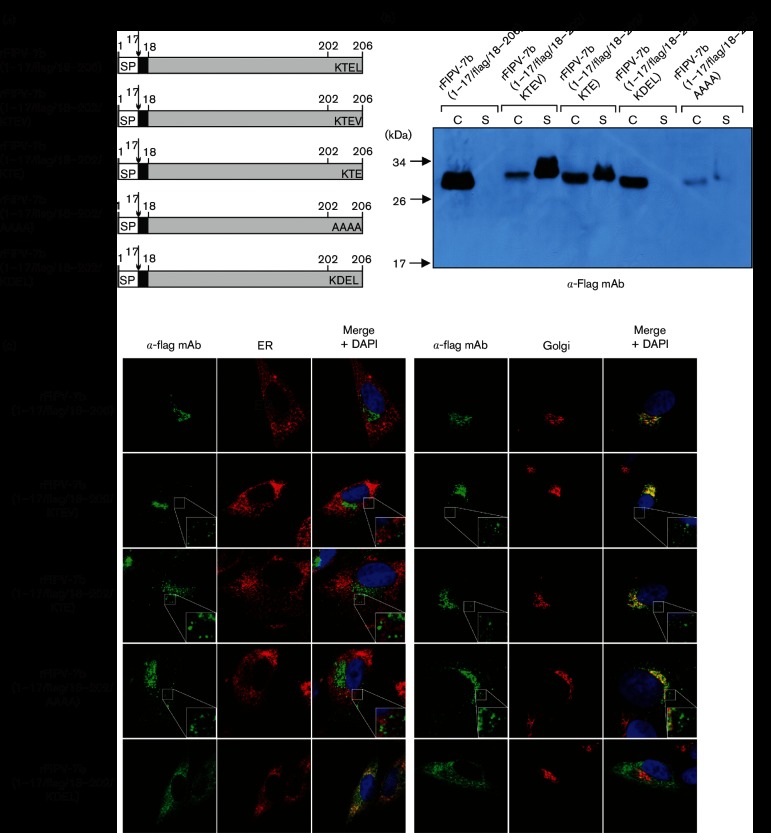
The role of the C-terminal tetrapeptide sequence in trafficking and secretion of the FIPV 7b protein. (a) Schematic representation of 7b proteins encoded by rFIPV-7b(1–17/FLAG/18–206), rFIPV-7b(1–17/FLAG/18–202/KTEV), rFIPV-7b(1–17/FLAG/18–202/KTE), rFIPV-7b(1–17/FLAG/18–202/AAAA) and rFIPV-7b(1–17/FLAG/18–202/KDEL). Black boxes represent the position of the FLAG tag in the respective 7b protein variants. The C-terminal amino acids of 7b are shown in the single-letter code; SP, signal peptide; numbers indicate amino acid positions in 7b. The arrow represents the signalase cleavage site. (b) CRFK cells were infected with rFIPV-7b(1–17/FLAG/18–206), rFIPV-7b(1–17/FLAG/18–202/KTEV), rFIPV-7b(1–17/FLAG/18–202/KTE), rFIPV-7b(1–17/FLAG/18–202/KDEL) and rFIPV-7b(1–17/FLAG/18–202/AAAA), respectively, with an m.o.i. of 0.1. Cells and cell culture supernatants were collected at 16 h p.i. Cell lysates (C) and cell culture supernatants (S) were separated by SDS-PAGE (10 %) under reducing conditions and analysed by Western blotting using anti-FLAG mAb (α-FLAG mAb). (c) CRFK cells were mock-infected or infected with rFIPV-7b(1–17/FLAG/18–206), rFIPV-7b(1–17/FLAG/18–202/KTEV), rFIPV-7b(1–17/FLAG/18–202/KTE), rFIPV-7b(1–17/FLAG/18–202/AAAA) and rFIPV-7b(1–17/FLAG/18–202/KDEL), respectively, with an m.o.i. of 1. Cells were fixed at 16 h p.i. and analysed by confocal laser-scanning microscopy. Immunofluorescence staining of 7b was performed using anti-FLAG mAb (α-FLAG mAb, green signal). Immunofluorescence staining of the endoplasmic reticulum (ER) (left panel, red signal) and the Golgi complex (right panel, red signal) is shown. Cell nuclei were stained with DAPI (blue signal). The inserts in the lower right corner represent an 8× magnification of the selected area.

The 7b protein was characterized earlier using a heterologous vaccinia virus/T7-based expression system [29]. The authors suggested that the C-terminal sequence KTEL of 7b represents a KDEL-like ER retention signal. Vennema and colleagues showed that a Leu-to-Val exchange in the KTEL motif abolished ER retention and facilitated secretion of the protein [29]. We therefore decided to study the effect of the KTEL motif on 7b subcellular localization in FCoV-infected cells in more detail. A set of recombinant viruses carrying substitutions in the 7b C-terminal KTEL motif were generated (Fig. 4a). First, a virus that contained a KTEV motif, rFIPV-7b(1–17/FLAG/18–202/KTEV), was generated. Second, we generated rFIPV-7b(1–17/FLAG/18–202/KTE), a virus that lacked the C-terminal Leu-206 residue in its 7b protein but retained a 3-residue KXD/E motif identified recently as a putative Golgi retention signal in plants and other eukaryotic systems [32]. Using this virus, we sought to answer the question of whether a KTE motif in 7b is sufficient for Golgi retention. Third, the entire C-terminal KTEL motif in 7b was replaced with AAAA, resulting in a recombinant virus designated rFIPV-7b(1–17/FLAG/18–202/AAAA).

CRFK cells were infected with the viruses in which the KTEL motif in 7b was altered and with rFIPV-7b(1–17/FLAG/18–206), respectively. Cells and cell culture supernatants were harvested at 16 h p.i. and analysed by SDS-PAGE followed by Western blotting using an anti-FLAG mAb (Fig. 4b). While 7b could be detected in all cell lysates, the 7b proteins with changes in the KTEL motif (KTEV, KTE and AAAA) could also be detected in cell culture supernatants, indicating that changes in the C-terminal KTEL motif of 7b led to secretion of the 7b protein. We also obtained a relatively weak signal for 7b in rFIPV-7b(1–17/FLAG/18–202/AAAA)-infected cells, possibly indicating rapid intracellular degradation of this particular protein. To corroborate possible effects of changes in the KTEL motif on 7b trafficking, confocal microscopy analyses were performed (Fig. 4c). The data revealed that 7b with altered KTEL motifs co-localized with the Golgi apparatus, but 7b signals could also be detected outside this compartment as dense spots in the cytoplasm of infected cells. Taken together, these data lead us to conclude that an intact KTEL motif at the C-terminus of 7b is important for efficient retention of this protein in the Golgi apparatus.

C-terminal KDEL motifs have previously been shown to act as ER retention signals [29, 33–35]. Using a vaccinia virus/T7-based system for the expression of 7b, Vennema and colleagues showed that a replacement of the KTEL motif with KDEL abolished 7b secretion and caused a complete retention of the protein in the ER [29]. To investigate whether the KDEL motif would cause a similar relocation of the 7b protein from the Golgi compartment to the ER in FCoV-infected cells, rFIPV-7b(1–17/FLAG/18–202/KDEL) was generated (Fig. 4a). Using Western blot analyses, the KDEL-containing 7b was only detected in cell lysates, but not in the supernatant (Fig. 4b). Furthermore, confocal microscopy analyses showed that 7b_KDEL co-localized exclusively with the ER (Fig. 4c). These observations are consistent with the findings of Vennema *et al*. [29].

Taken together, our results revealed that the authentic C-terminus of FCoV 7b is responsible for localization of the protein to the Golgi apparatus. Replacement of the KTEL motif with KTEV, KTE and AAAA, respectively, altered the trafficking of 7b and led to secretion of the protein, while a KTEL-to-KDEL replacement resulted in ER retention of the protein.

### Subcellular localization/trafficking of 7b following inhibition of protein synthesis

**Fig. 5.**
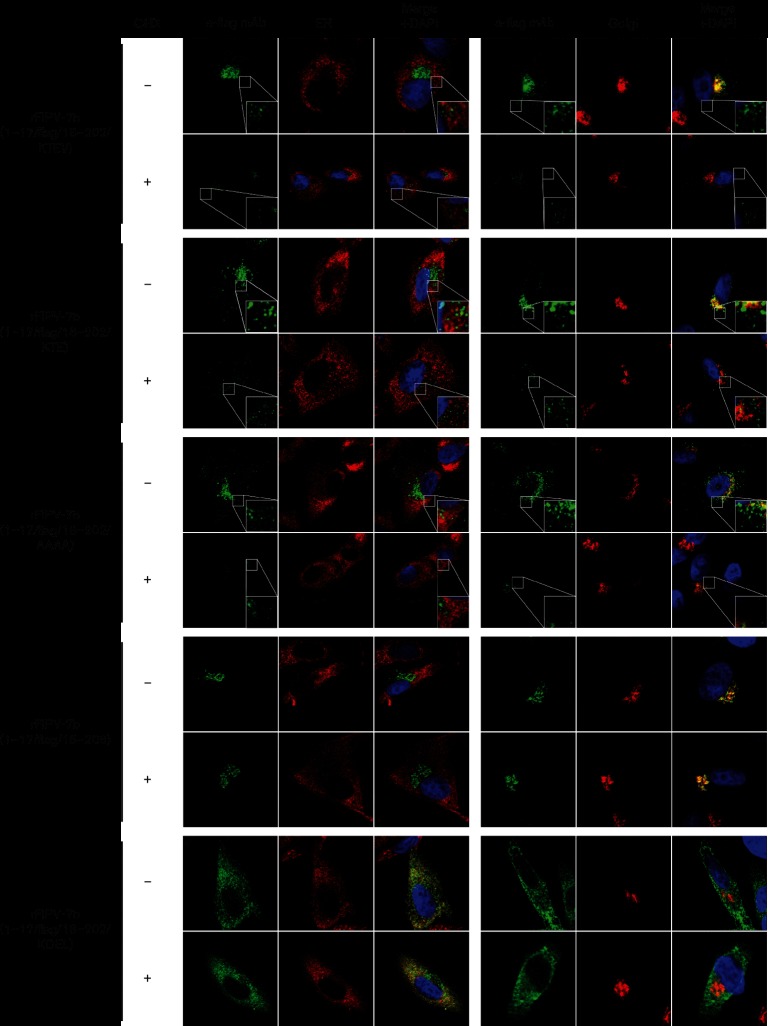
Effect of cycloheximide treatment on 7b trafficking. CRFK cells were mock-infected or infected with rFIPV-7b(1–17/FLAG/18–202/KTEV), rFIPV-7b(1–17/FLAG/18–202/KTE), rFIPV-7b(1–17/FLAG/18–202/AAAA), rFIPV-7b(1–17/FLAG/18–206) and rFIPV-7b(1–17/FLAG/18–202/KDEL), respectively, at an m.o.i. of 1. At 15 h p.i., the cells were treated with cycloheximide (+) or left untreated (−). At 17 h p.i., the cells were fixed and analysed by confocal laser-scanning microscopy. Immunofluorescence staining of 7b was performed using anti-FLAG mAb (α-FLAG mAb, green signal). Immunofluorescence staining of the endoplasmic reticulum (ER) (left panel, red signal) and the Golgi complex (right panel, red signal) is shown. Cell nuclei were stained with DAPI (blue signal). CHX, cycloheximide. The inserts in the lower right corner represent an 8× magnification of the selected area.

To monitor the intracellular localization and trafficking of already synthesized 7b protein, the localization of 7b was studied in virus-infected CRFK cells that were treated with cycloheximide to block cellular translation. The following viruses were used in this experiment: rFIPV-7b(1–17/FLAG/18–202/KTEV), rFIPV-7b(1–17/FLAG/18–202/KTE), rFIPV-7b(1–17/FLAG/18–202/AAAA), rFIPV-7b(1–17/FLAG/18–202/KDEL) and rFIPV-7b(1–17/FLAG/18–206). At 15 h p.i., the cells were treated with cycloheximide to block ongoing protein synthesis or were left untreated. At 17 h p.i., both cycloheximide-treated and untreated cells were fixed and 7b localization was analysed by confocal microscopy (Fig. 5). The subcellular localizations of 7b proteins with a C-terminal KTEV, KTE and AAAA sequence, respectively, were found to differ between cycloheximide-treated and untreated cells; in cells treated with cycloheximide, 7b did not co-localize with the Golgi, while weak signals of 7b could only be detected outside of this compartment. In contrast, there was no change in the subcellular localization of 7b with an intact KTEL (Golgi localization) or with a KDEL motif (ER localization) in cycloheximide-treated versus untreated cells. Similar results were also obtained in other feline cell lines (Fig. S1, available with the online Supplementary Material).

These data provide additional evidence to suggest that replacement of the KTEL motif with KTEV, KTE or AAAA reduces the efficiency of Golgi retention and, as a consequence, leads to secretion of this protein. Furthermore, the translation inhibition experiments suggest that the C-terminal KTEL motif in FCoV 7b functions as an efficient Golgi retention signal, while a single Thr-to-Asp substitution at aa position 204 was found to convert this Golgi retention signal (KTEL) into an ER retention signal (KDEL). This hypothesis is supported by the observation that even after 2 h of inhibition of cellular protein synthesis, the previously synthesized 7b_KDEL and 7b-KTEL proteins remained (exclusively) in the ER and Golgi compartments, respectively, rather than being transported along the exocytotic pathway.

## Discussion

In order to detect the accessory protein 7b in FCoV-infected cells, we first expressed 7b in bacteria and generated monoclonal antibodies (mAbs) specific for this protein. Unfortunately, the authentic glycosylated protein was not recognized by the mAbs, but only by its non-glycosylated precursor [30]. Using a recombinant virus that encodes 7b without the single N-glycosylation site, we were able to show that 7b is a non-secreted glycoprotein located in the Golgi apparatus. This result was unexpected, since previous studies using a vaccinia virus-based expression system in HeLa cells had suggested that 7b would locate to the ER and then be secreted into the tissue culture supernatant [29]. In order to investigate whether the lack of glycosylation is responsible for the observed differences, we constructed recombinant FCoVs to express FLAG-tagged forms of 7b. Computer-aided predictions suggested two potential signalase cleavage sites downstream of an N-terminal signal sequence. We determined the exact position of the signalase cleavage site in 7b by N-terminal sequence analysis (Edman degradation) of the proteolytically processed protein expressed in FIPV-infected cells and then used this information to introduce a FLAG tag immediately downstream of the experimentally confirmed cleavage site. Using viruses expressing these FLAG-tagged glycosylated or non-glycosylated forms of 7b, we found that the protein co-localized exclusively with the Golgi apparatus and was not secreted from infected cells. Furthermore, 7b was found to be resistant against Endo H treatment, suggesting that this protein was retained beyond the *cis*-Golgi compartment. The same results were not only observed with CRFK cells, but also with other cat cell lines (data not shown).

Next, we investigated whether the C-terminus of 7b is relevant for the subcellular localization of this protein. Using a recombinant virus that expressed a C-terminal FLAG-tagged 7b, we demonstrated that the protein was found in the entire cytoplasm as dense spots, as well as in the cell culture supernatant. This result is in accordance with a recent study, where a 7b–GFP fusion protein was expressed [28].

Interestingly, 7b contains a KDEL-like sequence at its C-terminus, namely KTEL. This motif was suggested to function as a KDEL-like ER retention signal [29]. By contrast, our data obtained with virus-infected feline cells strongly support the idea that the KTEL motif of FCoV 7b functions as a Golgi rather than an ER retention signal. Since this discrepancy was considered to be important, a panel of viruses carrying variations of the C-terminal KTEL motif in 7b was produced. Removal of the C-terminal residue (KTE), replacement of Leu with Val (KTEV) or exchange of the entire KTEL motif with four alanine residues (AAAA) caused a profound relocalization of 7b. Each of these three 7b protein variants localized to dense spots in the cytoplasm and was secreted into the cell culture supernatant. In contrast, the substitution of Thr with Asp (KDEL) resulted in complete ER retention of 7b with no secretion. Altogether, our experiments showed that the authentic C-terminus of FCoV 7b is required for the localization of this protein to the Golgi apparatus.

In previous studies, C-terminal KDEL and KTEL motifs in proteins with an N-terminal signal sequence have been linked to localization in the ER [29, 34, 36]. Proteins with such a sequence at their C-terminus apparently bind to KDEL receptors located in the *cis*-Golgi that trigger the retrieval of the complex to the ER [35, 37]. Based on this knowledge, it was surprising to see that the exchange of a single amino acid in the KTEL motif directs the localization of FCoV 7b to either the ER (KDEL) or the Golgi (KTEL). So far, three different KDEL receptor isoforms have been identified in human cells, with sequence identities ranging from 80 to 94 % [35, 38–41]. KDEL receptor isoforms are known to have distinct binding characteristics, with specific ligands being bound more efficiently than others [34, 41]. For human cells, it has been demonstrated that the KTEL motif at the C-terminus of proteins was recognized by KDEL receptors with almost the same efficiency as the KDEL motif and thereby conferred ER retention [29, 34]. Homologues of the human KDEL receptor isoforms also exist in other mammalian species. The sequences of feline KDEL receptor isoforms display 99, 98.6 and 95 % amino acid identities with their human counterparts. It is not known whether (and to what extent) differences between human and feline KDEL receptors affect the binding to specific ligands and, consequently, the retention/retrieval of specific proteins in the ER. We hypothesize that in the study performed by Vennema and co-workers, FCoV 7b was recognized by KDEL receptors in HeLa cells and consequently retained in the ER [29]. In contrast, the results of our experiments in feline cells suggest that the C-terminal KTEL motif of the FIPV 7b protein is not recognized by KDEL receptors. It would be interesting to test this hypothesis by expression of the authentic FCoV 7b protein in non-feline cells. Such experiments will be performed in the context of FCoV infection as well as with the authentic 7b protein alone. Moreover, the failure of cat cells to direct the 7b protein to the ER does not explain the retention of this protein in the Golgi. Based on our data, it is tempting to suggest that 7b secretion is prevented by interactions of the intact KTEL motif with a yet-to-be-identified viral or cellular protein. Furthermore, this study leads us to conclude that alterations of the KTEL motif (such as KTEV, KTE or AAAA, or the addition of a C-terminal FLAG tag) interfere with this interaction and thereby lead to secretion of 7b. Obviously, the identification of interaction partners of 7b in FCoV-infected cells is crucial to understand the molecular basis for 7b retention in the Golgi complex.

The retrograde protein transport system involving KDEL-binding proteins may provide a plausible explanation for the different observations regarding 7b protein secretion that were made in the present and a previous study [29]. It is known that overexpression of proteins may lead to a saturation of the KDEL receptor-mediated retention system, resulting in the secretion of proteins that are normally retained in the ER when they are produced at lower/physiological concentrations [33, 42]. Such an ‘overflow’ might explain why when the vaccinia virus/T7-based (over)expression system used by Vennema *et al*. [29] produced large amounts of FIPV 7b in HeLa cells, it resulted in a partial secretion of the protein (despite its efficient retention in the ER compartment). This earlier study also reported that 7b may be secreted from FIPV-infected FCWF cells. However, because this report did not provide information on the time point p.i. at which cell culture supernatants were collected for further analysis, it remains possible that 7b was released into the FCWF cell supernatant by cell damage caused by the FIPV infection at late points p.i. This would be fully consistent with our own observations, since we found that 7b can be detected in the supernatant of FCoV-infected cells at late time points p.i., when a cytopathic effect becomes detectable (data not shown).

The conservation of 7b homologues in feline and canine coronaviruses belonging to the species *Alphacoronavirus 1* suggests that the protein has host-specific functions. It would be interesting to establish the function of this protein in viral infections of the natural host. It is tempting to speculate that the Golgi-resident 7b protein helps to escape recognition by the host immune system, which in turn might facilitate the development of persistent infections by FCoVs. The generation of recombinant 7b-deficient FCoVs and their characterization in *in vivo* experiments will be required to test this hypothesis.

## Methods

### Cells and viruses

Crandell Rees feline kidney (CRFK) and *Felis catus* whole-foetus 4 (FCWF-4) cells were purchased from the American Type Culture Collection. Monkey kidney (CV-1) cells were purchased from the European Collection of Cell Cultures. The D980R cells were a kind gift from G. L. Smith, Imperial College, London, United Kingdom. The BHK-Tet/ON cells were a kind gift from N. Tautz, University of Luebeck, Germany. All cells were maintained in Dulbecco’s modified Eagle’s Medium (DMEM) supplemented with 10 % foetal calf serum (FCS), penicillin (100 U ml^−1^) and streptomycin (100 U ml^−1^) in 5 % CO_2_ at 37 °C. Recombinant FCoVs were propagated in CRFK, FCWF-4 and FC3TG cells. Recombinant vaccinia viruses were propagated, titrated, and purified as described previously [43–45].

### Plasmid construction and generation of recombinant vaccinia viruses

To generate recombinant vaccinia viruses containing a cDNA copy of the FCoV type II strain 79–1146 genome with genetic changes in the 7b gene, the previously described vrecFCoV-II vaccinia virus was used [45]. vrecFCoV-II derivatives were generated by two steps of homologous recombination and selection using *E.coli* guanosine-phosphoribosyltransferase (GPT) as described previously [43–47]. To construct vaccinia virus vrecFCoV-II-GPT-Δ7ab, where ORF7 was substituted with the GPT gene, plasmid pGPT-1-Δ7ab was used for vaccinia virus-mediated homologous recombination with vrecFCoV-II. Plasmid pGPT-1-Δ7ab is based on pGPT-1 [47] and contains sequences corresponding to nucleotides (nt) 27 651 to 28 151 and 29 083 to 29 355 of the serotype II FCoV strain 79–1146 downstream and upstream of the GPT gene, respectively. The recombinant GPT-containing vaccinia virus obtained after GPT-positive selection was then used for vaccinia virus-mediated homologous recombination with plasmids pDF1-pDF9 to generate recombinant vaccinia viruses containing the full-length cDNA of type II FCoV strain 79–1146 with genetically modified 7b genes.

Plasmid pDF1 was used to produce vrFIPV-7b(1–206/N68S). This plasmid is based on pGem-T (Promega) and contains nt 27 651 to 29 355 of the serotype II FCoV strain 79–1146. Additionally, nt 28 663 to 28 665 (AAT) were replaced with TCA, resulting in an Asn-to-Ser substitution at aa position 68 in 7b. To generate vrFIPV-FLAG-7b(1-206), plasmid pDF2 was used. It is based on pGem-T (Promega) and contains nt 27 651 to 29 355 of the serotype II FCoV strain 79–1146. Additionally, a FLAG tag coding sequence preceding the 7b gene (downstream of nt 28464) was introduced. Plasmid pDF3 was used to produce vrFIPV-7b(1–17/FLAG/18–206). pDF3 is based on pGem-T (Promega) and contains nt 27 651 to 29 355 of the serotype II FCoV strain 79–1146 and a FLAG tag coding sequence that was inserted downstream of nt 28512, corresponding to aa position 17 in the 7b protein. To generate vrFIPV-7b(1-206)-FLAG, plasmid pDF4 was used. This pGem-T (Promega)-based plasmid contains nt 27 651 to 29 355 of the serotype II FCoV strain 79–1146 and a FLAG tag coding sequence downstream of the 7b gene (3' to nt 29 079). Plasmid pDF5 was used to construct vrFIPV-7b(1–17/FLAG/18–206/N68S). This plasmid sequence corresponds to that of plasmid pDF1, but contains an additional FLAG tag coding sequence downstream of nt 28 512, corresponding to aa position 17 in the 7b protein. To generate vrFIPV-7b(1–17/FLAG/18–202/KTEV), plasmid pDF6 was used. The sequence of pDF6 corresponds to that of plasmid pDF3, except for a TTA-to-GTT codon replacement (nt 29 077 to 29079), resulting in a Leu-to-Val substitution at aa position 206 in 7b. Plasmid pDF7 was used to generate of vrFIPV-7b(1–17/FLAG/18–202/KTE). The sequence of pDF7 is identical to that of pDF3, except for nt 29 077 to 29 079 (encoding aa 206 in 7b), which were missing in pDF7. To generate vrFIPV-7b(1–17/FLAG/18–202/KDEL), plasmid pDF8 was used. The sequence of this plasmid is identical to that of plasmid pDF3, except for an ACT-to-GAT codon replacement (nt 29 071 to 29073), resulting in a Thr-to-Asp substitution at aa position 204 in the 7b protein. Plasmid pDF9 was used to construct vrFIPV-7b(1–17/FLAG/18–202/AAAA). The sequence of pDF9 was identical to that of pDF3, except for a replacement of nt 29 068 to 29 079 with a GCAGCTGCTGCA sequence, resulting in a 4-aa substitution at the carboxyl terminus of 7b (aa 203–206, Lys-Thr-Glu-Leu to Ala-Ala-Ala-Ala).

### Recovery of recombinant FCoV

To recover recombinant FCoVs, the genomic DNA from recombinant vaccinia viruses was isolated, cleaved with ClaI restriction enzyme (NEB), and used as a template for *in vitro* transcription. *In vitro*-transcribed RNA was then electroporated into BHK cells expressing the type II FCoV strain 79–1146 nucleocapsid protein (BHK-FCoV-N-II) as described previously [43–46]. The electroporated cells were co-cultivated with FCWF-4 cells and, after an incubation period of 48 h, the cell culture supernatant containing recombinant FCoVs was harvested for further analysis. The identities of the recombinant FCoVs were confirmed by reverse transcription polymerase chain reaction (RT-PCR) and sequence analysis.

### Immunoprecipitation and N-terminal Edman sequencing

rFIPV-7b(1–206/N68S)-infected CRFK cells from a 10 cm culture dish were collected at 24 h p.i. and resuspended in RIPA buffer [150 mM NaCl, 50 mM Tris, 1 % NP-40, 0.5 % Na-deoxycholate, 0.5 mM Pefabloc SC (Sigma-Aldrich), pH 8.0] containing 0.1 % sodium dodecyl sulfate (SDS). The lysate was incubated on a rotary shaker at 4 °C for 30 min and then centrifuged (13 000 r.p.m, 4 °C, 30 min). Anti-7b mAb 14D8 [30] was added to the supernatant and incubated on a rotary shaker for 1 h at 4 °C. Protein G Sepharose (GE Healthcare) was added to the sample and incubated for another 1 h. Following centrifugation and washing, the pellet was mixed with protein loading buffer (2 % SDS, 10 % glycerol, 5 % β-mercaptoethanol, 0.01 % bromophenol blue) and incubated for 10 min at 96 °C. After a brief centrifugation step, proteins in the supernatant were separated in a 10 % tricine-polyacrylamide gel [48]. Following transfer to a PVDF membrane (Roth), proteins were visualized by Ponceau staining and the band corresponding to the non-glycosylated form of 7b (24 kDa) was excised and subjected to amino-terminal sequence analysis by automated Edman degradation on a gas-phase sequencer, Procise model 491 (Applied Biosystems). Phenylthiohydantoin derivatives of amino acids were identified by an online analyser, model 140C (Applied Biosystems).

### SDS-PAGE and Western blotting

CRFK cells were infected with rFIPVs (m.o.i. of 0.1). At 16 h p.i., the cells were suspended in RIPA buffer containing 2 % SDS and sonicated. Where indicated, the cell lysates were treated at 37 °C with specific glycosidases, PNGase F or Endo H (NEB), according to the manufacturer’s protocol. For SDS-PAGE analysis, the samples were mixed with protein sample buffer, heated for 5 min at 94 °C and loaded on a 10 % tricine-polyacrylamide gel [48]. For Western blot analysis, proteins separated by SDS-PAGE were transferred onto a nitrocellulose membrane (GE Healthcare). The membrane was blocked, washed with PBST (phosphate-buffered saline containing 0.1 % Tween-20) and incubated with primary antibody for 1 h at room temperature. To detect 7b protein, anti-7b mAb 14D8 [30] or anti-FLAG mAb (Sigma) was used. After washing with PBST, the membrane was incubated with horseradish peroxidase-conjugated goat anti-mouse antibody (Dianova) for 1 h at room temperature. Bound antibodies were visualized with chemiluminescent reagent (Western Lightning Plus-ECL, PerkinElmer).

### Indirect immunofluorescence assay

CRFK cells were cultured on sterile cover slips in 24-well plates and infected with rFIPVs at an m.o.i. of 1 or mock-infected. At 16 h p.i., the cells were fixed with 2 % paraformaldehyde for 20 min at room temperature (RT). If indicated, protein synthesis was inhibited using cycloheximide (CHX) (Sigma). CHX was added to the cell-culture media at 15 h p.i. for 2 h. At 17 h p.i., the cells were fixed, washed with PBS, permeabilized with 1 % Triton X-100 in PBS, and washed again with PBS. Residual binding sites were blocked with 1× Roti-ImmunoBlock (Roth) for 10 min at room temperature. Cells were incubated with appropriate primary antibodies for 1 h at room temperature. Anti-FLAG mAb (Sigma) was used to detect FLAG-tagged 7b protein. For the detection of the endoplasmic reticulum (ER), anti-protein disulfide isomerase antibody (Sigma) was used. The Golgi apparatus was detected using anti-Giantin antibody (Abcam) according to the manufacturer’s recommendations. Cells were washed with PBS and subsequently incubated with appropriate secondary antibodies, Alexa Fluor 488 goat anti-mouse (Invitrogen) or Alexa Fluor 594 goat anti-rabbit (Invitrogen), for 1 h at room temperature according to manufacturer’s instructions. DNA was stained with 4′,6-diamidino-2-phenylindole (DAPI). Following three washing steps with PBS, cover slips were mounted with Mowiol (Sigma) containing anti-fading reagent DABCO [1,4-diazabicyclo(2.2.2)octane, Roth]. The immunofluorescence staining was visualized by confocal laser-scanning microscopy (Leica TCS SP5).

## Supplementary Data

340 Supplementary File 1
