# Supplementary material for: Identification and characterization of a Golgi retention signal in feline coronavirus accessory protein 7b
Source: J Gen Virol. 2017 Jul 31;98(8):2017–29. doi: 10.1099/jgv.0.000879 (PMC7212014; doi:10.1099/jgv.0.000879)
Supplement: Supplementary file 1 [file jgv-98-2017-s001.pdf]

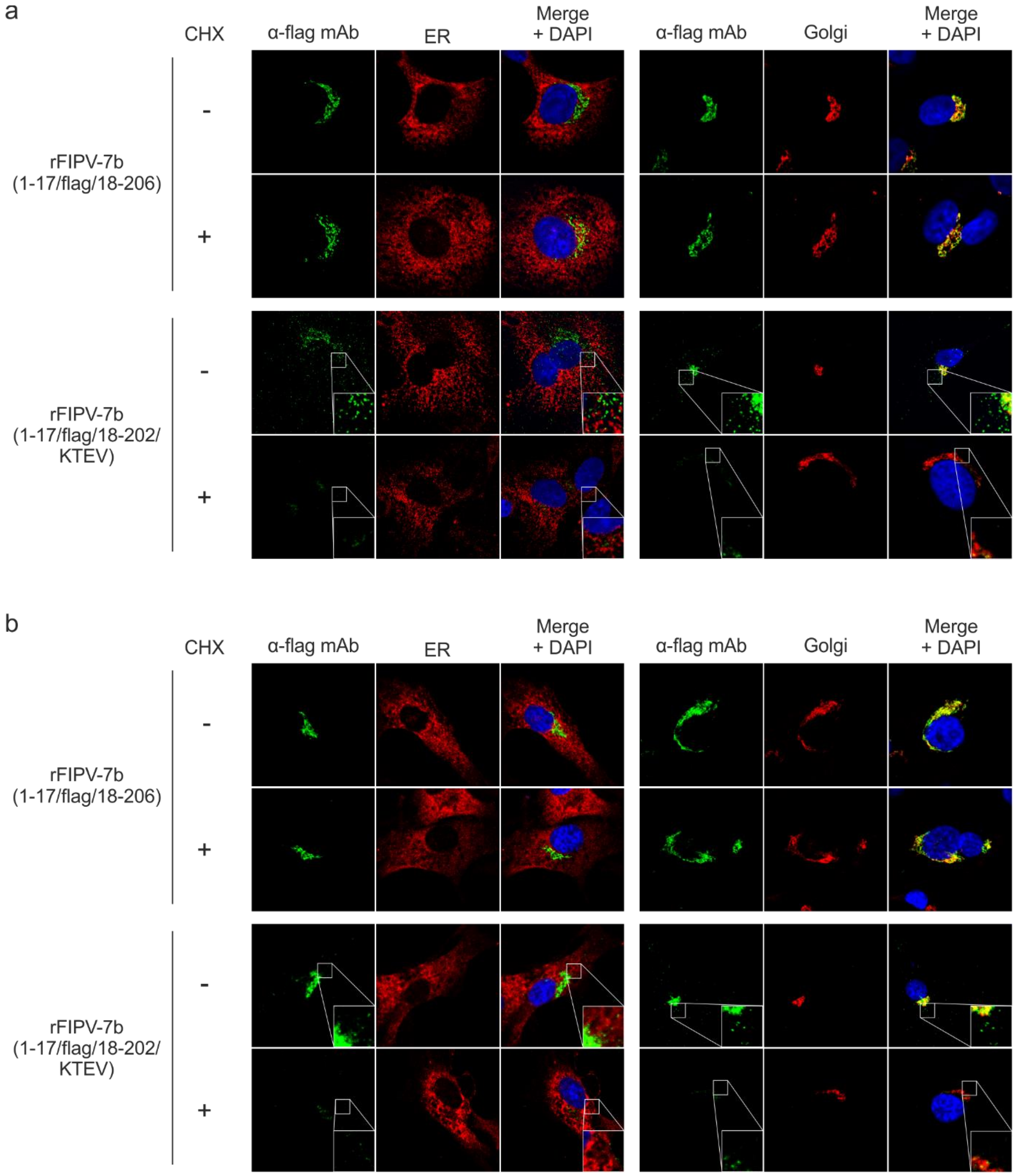

Fig. S1. Effect of cycloheximide treatment on 7b protein trafficking in FCWF-4 and Fc3Tg cells infected with FIPV. FCWF-4 **(a)** or Fc3Tg **(b)** cells were infected with rFIPV-7b(1-17/flag/18-206) and rFIPV-7b(1-17/flag/18-202/KTEV), respectively, at an MOI of 1. At 8 h p.i., the cells were treated with cycloheximide (+) or left untreated (-). At 10 h p.i., the cells were fixed and analyzed by indirect immunofluorescence assay. 7b protein was immunostained using anti-Flag® M2 (α-flag mAb, green signal) and endoplasmic reticulum (ER) (left panel, red signal) and Golgi apparatus (right panel, red signal) were stained using the antibodies described in Material and Methods. Cell nuclei were stained

using DAPI (blue signal). CHX, cycloheximide. Boxes in the lower right corners represent 8x magnifications of the selected areas.
